# A Critique on Maternal Insanity: A Case of Postpartum Psychosis and Infanticide

**DOI:** 10.1002/ccr3.72534

**Published:** 2026-04-15

**Authors:** Abdullah Hassan, Habib Ullah, Abdullah Wahdan Yahya, Arooma Sagheer, Bilal Hassan, Sardar Noman Qayyum, Rafiullah Hotak

**Affiliations:** ^1^ Ayub Medical College/Ayub Teaching Hospital Abbottabad Pakistan; ^2^ MTI, Ayub Teaching Hospital Abbottabad Pakistan; ^3^ Khyber Medical College/Ayub Teaching Hospital Abbottabad Pakistan; ^4^ Ayub Teaching Hospital Abbottabad Pakistan; ^5^ Bacha Khan Medical College Mardan Pakistan; ^6^ Nangarhar University Faculty of Medicine Jalalabad Afghanistan

**Keywords:** mental health, obstetrics/gynecology, perinatal medicine, psychiatry

## Abstract

Postpartum psychosis is a rare but severe psychiatric crisis that generally occurs during the early postpartum period and requires immediate attention. This case report focuses on an under‐recognized case of postpartum psychosis and its consequences. The patient, a 28‐year‐old mother, presented with symptoms 6 months after her uncomplicated childbirth. She had no prior history of psychiatric illnesses. Despite this, due to poorly understood stressors, she developed severe psychotic symptoms such as hallucinations, delusions of guilt, and intense emotional dysregulation. Her condition led to an infanticide and an attempt on the lives of her two older children before she attempted suicide. Her detailed clinical assessment revealed a severe mood disorder with psychotic and manic characteristics (PANSS = 69, YMRS = 8). The patient was treated with sessions of electroconvulsive therapy and antipsychotic trials. While her symptoms gradually improved, the aftermath of her actions left her dealing with unbearable grief, legal consequences, and societal stigma, giving us proof of the far‐reaching consequences of the initial misdiagnosis and non‐cooperation of the family toward early hospitalization, ultimately leading to delayed critical intervention. This case tries to understand the triggers of this mental state better and calls attention to conventional limitations in perinatal mental health awareness and insists on the prolongation of maternal mental health surveillance beyond the usual postpartum period. It is further discussed how more efficient screening tools (e.g., PPSS) in antenatal obstetric care setups and culturally appropriate family education need to be implemented, especially in marginalized populations where high family‐related psychosocial stressors can be a clinical risk. Postpartum psychosis and accompanying circumstances can burden patients with ethical and legal dilemmas surrounding their actions during the psychotic episodes. We also emphasize the need for rehabilitation of patients and their families, concluding with a focus on a multidisciplinary approach to address the outcomes of this disease.

## Key Clinical Message

Postpartum psychosis may present several months after childbirth, resulting in a risk to both the maternal and infant life if not timely identified. This case underscores the necessity to have a prolonged postpartum psychiatric observation, culturally appropriate family education, and timely multidisciplinary intervention to avoid tragic outcomes.

## 1 Introduction

Louis‐Victor Marcé, a nineteenth‐century psychiatrist, writes in his monograph, “Some women in whom depression is not strong enough to rob the mind of all lucidity show a cold‐bloodedness in the execution of their plans and a cleverness that can't always be outsmarted” [1].

Childbirth is a vulnerable time in a woman's life. She goes through multiple physical and emotional challenges. She must deal with lack of sleep and severe exhaustion, bodily and hormonal changes, and above all, she has to take up the role of a mother and take on the responsibility of bringing up a new life. Most women experience some psychological changes during these days, such as mood swings, anxiety, and mild depression. Conversely, these changes can also take the form of extreme conditions such as full‐blown psychotic episodes.

Postpartum psychosis/Puerperal psychosis is a rare and severe form of mental illness that occurs suddenly after childbirth. The literature on onset is very vague, using terms like “within a few days,” “2 to 6 weeks after,” etc., but there is evidence for cases occurring as late as 9 months after delivery [2]. Symptomatology shows cognitive disorientation in women with childbearing onset‐related psychosis that is not seen in women with non‐childbearing onset affective psychosis [3]. This means that there is a blend of symptoms, including extreme confusion, mania, depression, paranoia, odd behavior, delusions of persecution and reference, hallucinations, and many others, giving a picture that has been referred to in the psychiatric text as postpartum psychosis. Although the absolute incidence of first‐time presentation of postpartum psychosis is only 0.89–2.6 per 1000 births [4], the increased associated risk of maternal suicide makes it a psychiatric emergency. Additionally, it is also associated with infanticide driven by various motives [5]. It is estimated that 4% of women suffering from postpartum psychosis commit infanticide [6]. Including purpuric illnesses in literature as separate nosological entities has been debated for decades [7]. ICD‐10 categorizes postpartum psychosis under severe mental and behavioral disorders associated with puerperium, not classified elsewhere, whereas DSM‐5 has no separate category for it. Therefore, current diagnostic standards inadequately recognize it and do not consider it a distinctive nosological entity. This might have contributed to misdiagnosis and low awareness among clinicians regarding its dangers.

The causes and triggers of postpartum psychosis are poorly understood. Though considered a feature of bipolar affective disorder or a characteristic of schizophrenia, making a previous history of psychiatric illness the single most important risk for the disorder, studies have shown that 50% of the cases experience the first episode of postpartum psychosis with no history of psychiatric illness [8]. Jones et al. found a much higher incidence in women with a strong family history, suggesting a genetic influence running in selected families. The current view suggests a link between the sudden loss of maternal hormones at birth and its association with mental instability [9]. According to several other studies, primiparity [1], lack of proper social support [10], absence of husband during delivery, poor socioeconomic status, young age of mother, obstetric and neonatal complications [11], longer interval between preceding pregnancy and the latest [12] are some of the other important risk factors.

This case reveals a tragic presentation of a woman in her late 20s who suffered from an acute puerperal manic episode of psychosis that may have contributed to the murder of her newborn, an attempt on the life of two of her other children, followed by her suicidal attempt. This case is rare since the episode occurred 6 months postnatal, while most, if not all, cases of postpartum psychosis occur early on, about 6 weeks after delivery. This case will further focus on raising awareness, early detection, management, and recommendations for preventing such cases so that such future events can be avoided.

## 2 Case Presentation/History

The patient is a married woman in her late 20s, a mother of 4, who presented to the emergency department of our hospital 8 months after the birth of her fourth child for a suicide attempt. First, she ingested roughly 100 tablets of Alprazolam, Paroxetine, and Olanzapine. Following this, she attempted to take the lives of her three children. She slit the throat of her 8‐month‐old newborn with a kitchen knife, and the newborn died after being fatally injured. She then attempted the same on her two older sons, aged 6 years and 2 and a half years, who survived after emergency surgical intervention. Upon admission, the patient was initially in a confused state and unable to act out the act of infanticide. However, she had a vague, fragmented recollection of events around her attempts on the lives of older children and her own.

One can find noteworthy psychosocial stressors in the patient's background story. She was born and raised in a conservative middle‐class family in the suburbs of Abbottabad. She got married at the age of 22 and completed her bachelor's degree in Computer Science at the age of 24. Her marriage was to her cousin, and she had to settle within a joint family system, which led to a life fraught with domestic strife due to family conflicts. Her first three pregnancies were uncomplicated, and her recent birth was medically unremarkable with no reports of gestational diabetes, chronic hypertension, preeclampsia, or eclampsia. However, she experienced severe stress throughout pregnancy and frequently became agitated, but she was never screened for any psychiatric illness.

The patient delivered a 3 kg healthy male baby via normal vaginal delivery at a tertiary care hospital with no reported neonatal complications. Approximately for the 6th month postpartum, she took good care of her child and was psychiatrically asymptomatic. However, at month six, she began to develop insomnia, emotional overwhelm, and an inability to handle childcare duties. She had recurrent intrusive thoughts of self‐harm and harming her children. Over the subsequent weeks, she displayed suicidal and homicidal behaviors, resulting in multiple non‐fatal attempts, including a failed attempt at poisoning with high doses of medication added to the food of the family and another attempt at suffocation of her children, which she abandoned due to moral constraint. Upon this, her family decided to seek psychiatric consultation. She was diagnosed with postpartum psychosis, and pharmacological treatment was initiated. However, upon no significant improvement, she was medically advised for inpatient admission. Despite the emphasis on the urgency of hospitalization by the psychiatrists, the family declined. Thereafter, she made another aborted attempt involving household gas exposure with the intention of suffocating the family. The index event occurred approximately 8 months postpartum, resulting in infanticide, attempted filicide, and a suicide attempt.

## 3 Differential Diagnosis, Investigations and Treatment

After admission to the psychiatry ward, the patient was observed to have manic symptoms such as an elated mood with extreme irritability. She had racing thoughts, rapid speech, and switched from one topic to another. She displayed impulsivity, such as the decision to divorce her husband or make an attempt to take his life. She also had notable psychotic features, such as a delusion of guilt regarding her fourth child's birth. She held a conviction that the child would end up being a societal threat, culminating in the belief that infanticide was necessary. The patient's history was unremarkable for substance use or any prior neurological disease. All the previous medical records lacked any evidence that could indicate metabolic, endocrine, or toxicological abnormalities that might have resulted in her current presentation. A medical evaluation was performed to rule out other possible etiologies. A toxicology screening was performed that showed no signs of substance abuse or intoxication. Furthermore, a neurological examination was also performed, which showed neither features of delirium nor any focal deficits. Other than that, the routine laboratory investigations, including Complete Blood Count, Electrolytes, Liver and Renal Function Tests, and Thyroid Profile, were also very much within the normal limits. Hence, in the absence of any neurological, medical, or endocrine comorbidities, she was diagnosed with postpartum psychosis with manic and psychotic features. The patient's family history is largely unremarkable for psychiatric illnesses, except for an episode of postpartum psychosis in her younger sister, which was successfully treated. On psychological assessment, she scored 12 out of 17 on the Hamilton Rating Scale for Depression (HAM‐D) and 8 out of 11 on the Young Mania Rating Scale (YMRS). On the Brief Psychiatric Rating Scale (BPRS), she had elevated scores for psychotic symptoms, including hallucinations and delusions. Her total score on the PANSS rating scale was 69, with a score of 33 on the positive rating subscale, 18 on the negative rating subscale, and 28 on the general psychopathology subscale.

For treatment, we initiated with a combination of antipsychotics and electroconvulsive therapy (ECT). In total, 12 ECT sessions were done, which showed significant improvement in her irritability and excessive talking. Her highly energetic spells subsided, and she no longer had ideas of self‐harm or infanticide. As for pharmacological intervention, the patient was started on Olanzapine and Haloperidol to manage her psychotic symptoms. Initially, Lithium was prescribed as a mood stabilizer; however, she was switched to Sodium Valproate as she appeared to be a suboptimal candidate for Lithium therapy on monitoring. Clonazepam was also added to manage agitation and insomnia.

## 4 Conclusion and Results (Outcome and Follow‐Up)

After noticing considerable improvement and with the consultation of her family, the patient was informed of the details about the traumatic events under an inpatient setting. The patient also engaged in supportive psychotherapy sessions to help her manage stress, better understand her condition, and make plans for the future. She was discharged after 6 weeks and transitioned to outpatient care, where she continues to receive psychotherapy sessions and follow‐ups. Her medication regimen was adjusted. The patient has been carefully replanted in her family life with supervision. Resources were provided for postnatal support, and her surviving children are now receiving appropriate care. Her long‐term plan includes psychiatric monitoring for any recurrence and to ensure complete adherence to her treatment plan. Table 1 summarizes the timeline of the symptoms.

**TABLE 1:** The chronological progression of symptoms and interventions.

Timeline of symptoms	Clinical events
Delivery (0 months)	Normal vaginal delivery of a healthy male infant
0–6 months postpartum	Psychiatrically asymptomatic
6 months postpartum	Onset of insomnia, emotional overwhelm
6–7 months postpartum	Multiple non‐fatal attempts including food adulteration and suffocation
~7 months postpartum	Psychiatric consultation, diagnosis of postpartum psychosis, advised for hospitalization
~7.5 months postpartum	Aborted attempt involving household gas exposure
~8 months postpartum	Infanticide, filicide, and suicide attempt leading to emergency admission

## 5 Discussion

This case report presents a relatively unfamiliar instance of postpartum psychosis where the onset of the disease was 6 months after delivery. Not only is the disease itself uncommon, but its progression to the extremes of maternal filicide of three children is a sporadic occurrence. This tragic case underscores the necessity to keep monitoring the mental health of the mothers months after the expected period of onset. The discussion can be deepened by further exploring possible interventions that may not have been previously addressed.

First, to assist with the early detection of postpartum psychosis, standardized screening tools should be implemented. Traditionally, for the assessment of maternal mental health, the gold standard has been clinical interviews, but they are both resource‐intensive and time‐consuming, especially in outpatient settings. Tools such as the Edinburgh Postnatal Depression Scale (EPDS) and the Mood Disorder Questionnaire can help detect postpartum psychosis in women during both antepartum and postpartum routine check‐ups [13]. Since distinguishing between postpartum depression and postpartum psychosis can be confusing, incorporating tools such as the Postpartum Psychosis Screening Scale (PPSS) can aid clinicians with quicker diagnosis [14]. Despite their clinical utility, however, the implementation is compromised by resource limitations, poor psychiatric infrastructure, time constraints in obstetric settings, and inadequate training among primary healthcare workers. Consequently, due to these hurdles, a shift towards high‐risk focused screening, targeting patients with prior psychiatric history or significant psychosocial stressors, may be more practical [15]. In addition to this, the incorporation of antenatal psychiatric services focused on improving communication skills between partners and between family members in high‐risk patients can reduce the overall likelihood of severe postpartum complications.

Though most clinicians are aware of the fundamental link between postpartum psychosis and a history of psychiatric illness, several other equally important risk factors can go unnoticed. Antenatal maternal complications like antepartum hemorrhage, preeclampsia, eclampsia, chronic hypertension, and delivery by caesarean section can be contributors to maternal mental instability [16]. Other well‐established risk factors include family history, primiparity [1], inadequate psychosocial setup [10], absence of husband during delivery, poor socioeconomic status, young mother [11], and longer gap between consecutive pregnancies [12]. Therefore, medical professionals, from physicians and obstetric staff to midwives, should closely monitor and assess such individuals. When necessary, timely intervention should be taken to refer or admit them to inpatient psychiatric care. The transition from inpatient to outpatient should be carefully managed to ensure that the patient receives satisfactory support and medication management after discharge. Structural follow‐up protocols to make adjustments to treatment plans based on a patient's evolving needs can significantly improve long‐term outcomes. These regular check‐ins should also continue for several months, regardless of whether the symptoms are present in high‐risk populations, to ensure adequate psychiatric monitoring.

Second, the role of family education and support cannot be emphasized enough. As demonstrated in this case, the family was unaware of the severity of her disease and took it lightly till it escalated into a crisis. Additionally, the patient's background, including her marital and socioeconomic status, likely contributed to her mental decline. Thus, educating families about the possible postpartum mental issues in new mothers can enhance their ability to provide better support, which can help mitigate the stressors that otherwise may exacerbate the symptoms and assist with early detection of danger signs. There should also be newborn‐maternal attachment initiatives with the help of the family. This can be done through family counseling, community support groups, and public seminars [17].

Third, this case also highlights the interplay of forensic psychiatry, ethics, and rehabilitation in association with severe postpartum psychosis. Acts committed during an acute psychotic state complicate the traditional frameworks of criminal responsibility, especially in societies where mental health is still highly stigmatized. Furthermore, the psychological trauma faced by the surviving children is immense, demanding rehabilitation between them and their affected mother. Along with this, the patient is bound to face momentous legal, social, and moral consequences. Additionally, delayed recognition and refusal of family for hospitalization raise concerns of legal negligence. In the events where the illness leads to these outcomes, inpatient family integration therapy can play a major role in the post‐trauma healing process between the patient and her family, especially her children [18].

There should be public campaigns to better educate the masses about the severity of mental illnesses and their possible consequences. In the absence of these interventions, the emotional distress from criminal charges, public judgment on the mother's character and intent, and social stigmas can significantly interfere with the mental and social recovery of the patient.

## 6 Conclusion

Overall, this report presents a rare case of late‐onset postpartum psychosis that progressed to severe complications of infanticide and attempted suicide. We highlight the importance of ongoing monitoring of high‐risk patients beyond the typical timeframe. The case illustrates the need for a multifaceted approach to postpartum psychosis with training of medical professionals to apply newer screening methods and timely refer high‐risk patients for inpatient management. While developed countries may have well‐established protocols regarding maternal health education, in developing regions like Pakistan, there is still much room for comprehensive social and family education of high‐risk patients. Therefore, by increasing awareness about the symptoms of postpartum psychosis, we can allow patients and their families to seek help on time, thereby reducing the stigma associated with mental health issues during the perinatal and postnatal period, and in the long run, we can prevent the disease progression to severe complications.

## Author Contributions

**Abdullah Hassan:** conceptualization, investigation, validation, writing – original draft. **Rafiullah Hotak:** conceptualization, writing – original draft, writing – review and editing. **Habib Ullah:** conceptualization, methodology, writing – original draft. **Abdullah Wahdan Yahya:** investigation, writing – original draft. **Arooma Sagheer:** validation, writing – original draft, writing – review and editing. **Bilal Hassan:** validation, writing – original draft. **Sardar Noman Qayyum:** writing – original draft, writing – review and editing.

## Funding

The authors have nothing to report.

## Consent

Written informed consent was obtained from the patient for publication of this case report. A copy of the written consent is available for review by the editor‐in‐chief of this journal on request.

## Conflicts of Interest

The authors declare no conflicts of interest.

## Data Availability

The data used to support the findings of this study are included within the article.
